# Assessment of Cosmetic Properties and Safety of Use of Model Washing Gels with Reishi, Maitake and Lion’s Mane Extracts

**DOI:** 10.3390/molecules27165090

**Published:** 2022-08-10

**Authors:** Aleksandra Ziemlewska, Magdalena Wójciak, Kamila Mroziak-Lal, Martyna Zagórska-Dziok, Tomasz Bujak, Zofia Nizioł-Łukaszewska, Dariusz Szczepanek, Ireneusz Sowa

**Affiliations:** 1 Department of Technology of Cosmetic and Pharmaceutical Products, Medical College, University of Information Technology and Management in Rzeszow, Sucharskiego 2, 35-225 Rzeszow, Poland; 2 Department of Analytical Chemistry, Medical University of Lublin, AlejeRaclawickie 1, 20-059 Lublin, Poland; 3 Chair and Department of Neurosurgery and Paediatric Neurosurgery, Medical University of Lublin, 20-090 Lublin, Poland

**Keywords:** mushroom extracts, washing gel, bioactive compounds, skin cells, irritant potential

## Abstract

Natural cosmetics are becoming more and more popular every day. For this reason, this work investigates the properties of mushroom extracts, which are not as widely used in the cosmetics industry as plant ingredients. Water extracts of *Grifolafrondosa* (Maitake), *Hericiumerinaceus* (Lion’s Mane) and *Ganoderma lucidum* (Reishi) were tested for their antioxidant properties, bioactive substances content, skin cell toxicity, ability to limit TEWL, effect on skin hydration and pH, and skin irritation. Our research showed that Maitake extract contained the highest amount of flavonoids and phenols, and also showed the most effective scavenging of DPPH and ABTS radicals as well as Chelation of Fe^2+^ and FRAP radicals, which were 39.84% and 82.12% in a concentration of 1000 µg/mL, respectively. All tested extracts did not increase the amount of ROS in fibroblasts and keratinocytes. The addition of mushroom extracts to washing gels reduced the irritating effect on skin, and reduced the intracellular production of free radicals, compared with the cosmetic base. Moreover, it was shown that the analyzedcosmetics had a positive effect on the pH and hydration of the skin, and reduced TEWL.

## 1. Introduction

Currently, natural cosmetics are becoming more and more popular. They contain compounds mainly derived from plants, instead of synthetic substances which are often harmful [1,2]. The adverse health effects of these substances are primarily associated with their allergenic and irritating effects, as well as the harmful effects of heavy metals on the entire body. Their presence in cosmetic preparations often leads to atopic dermatitis, erythema, rashes and other allergic reactions, which is often the result of the additive effect associated with the presence of the same ingredient in many cosmetic products [3,4,5]. Currently, there are many studies describing various plant species that have a beneficial effect on skin and hair, and, therefore, are used in the production of cosmetics. However, it is not only plants that have this effect. Mushrooms also contain many bioactive compounds such as flavonoids, phenolic compounds, terpenes, polysaccharides, and fatty acids [6,7]. Thanks to their presence, mushroom extracts show anti-cancer, anti-inflammatory, antioxidant and antimicrobial properties [6,8,9,10,11,12,13]. Both fruiting bodies and mycelia have such features, and due to them, are used in cosmetology [6,14,15]. Due to their antioxidant properties and ability to absorb UV radiation, they are added to anti-aging cosmetics [15]. Mushroom extracts show a moisturizing and lightening effect on the skin, which also makes them useful in the cosmetics industry [15]. Mushrooms withanti-inflammatory and antimicrobial effects can be found in preparations for sensitive and problematic skin [14,15], however, cosmetics based on mushroom extracts are still not as popular as, for example, those containing compounds of plant origin. This is the reason for further research in this direction.

In these studies, we tested water extracts of three mushroom species: *Grifolafrondosa*, *Hericiumerinaceus* and *Ganoderma lucidum*. *G. frondosa*, also known as Maitake, is commonly used in Japan and China in herbal medicine [16]. It belongs to the Basidiomycota division and Polyporales family. The fruiting bodies of these fungi are bush-branched, composed of many wavy caps growing from a common trunk. The edges of the caps are uneven, cut, and wrinkled [17]. It has been proven that Maitake mushrooms have antitumor, antioxidant, and anti-diabetic activities. These properties are due to the high content of polysaccharides, as well as the presence of other compounds such as phenolic compounds [16]. *Hericiumerinaceus*, also known as Lion’s Mane, is a commonly known mushroom in Japan and China, used in alternative medicines and as a food supplement. It be-longs to the family Hericiaceae, order Russulales, and class Agaricomycetes. Due to the presence of polysaccharides, phenolic compounds and other biologically active substances, *H. erinaceus* shows antimicrobial, antioxidant, and anti-aging activity. Additionally, it has been shown that the water extract of *H. erinaceus* promotes wound healing [18,19]. *Ganoderma lucidum*, also called Reishi, is a species belonging to the class Agaricomycetes and Ganodermataceae family. In Korea, China, and Japan, it is a well-known medicinal mushroom which exhibits antioxidant, antiperoxidative, antibacterial, antitumor, antimutagenic and anti-inflammatory activity. For this reason, it is used as a food additive, in pharmaceutical products, and also as an ingredient in cosmetics [20,21,22].

Due to the high concentration of polysaccharides in the dry matter of mushrooms, mushroom extracts may be a valuable raw material for cleansing cosmetics. These groups of cosmetic products are used by consumers in high amounts, many times a day. The biggest disadvantage of cleansing cosmetics is the potential to cause skin irritation, which is induced by surfactants—the main ingredients of this group of cosmetics [23,24,25]. Surfactants have the ability to interact with skin proteins, leading to their denaturation and washing out of the skin. They are the main cause of skin irritations appearing after using cosmetics such as shower gels, shampoos, and hand soap liquids [23,24,26,27,28,29,30]. The force of irritant potential depends on the type of surfactants used in cosmetic formulations. Anionic surfactants have the strongest ability to induce an irritant effect because of their strong, electrostatic interaction with skin proteins. The irritant effect also depends on the form of surfactant molecules in the bulk phase of the products. The strongest irritant properties have been proven for surfactant monomers that have been identified, in the highest concentration (below critical micelle concentration (CMC)). Irritant potential is lower above CMC because of the formation of micelle aggregates. Monomers, as individual molecules of surfactants, are characterized by a smaller size than micelles, may penetrate deeper into the skin, and the probability of their interaction with skin proteins is higher. After micelles’ formation, the concentration of monomers in the solutions of surfactants is very low, and they arise as the result of the disintegration of thermodynamically unstable micelles [23,25,30,31]. There are many methods to decrease the irritant potential of surfactants. One of the most popular methods, and used in industrial practice, is the use of mixtures of various types of surfactants (for example, anionic, nonionic and amphoteric), which increases the stability of micelles and reduces the amount of monomers released from micelles to the bulk phase of a surfactant’s solution. The second methodis the use of high molecular weight substances such as natural and synthetically derived polymers. Such substances create so-called “necklace structures” with micelles (micelles connected with the active centers of polymer chains) which leads to anincrease in the micellestability [31,32,33,34]. Mushroom polysaccharides, proteins and carbohydrates, as natural polymers, may interact with surfactant micelles and cause an increase in their stability and, in fact, lead to a reduction in irritant potential.

In order to investigate the properties of the water extracts of mushrooms, a cytotoxicity test was performed on skin cells. Furthermore, the content of flavonoids and phenolic compounds was determined, and the antioxidant properties of the examined extracts were examined. Obtained extracts were used as the active ingredient of model body wash gels, in order to increase their safety of use. For the obtained cosmetics containing the analyzed extracts and the reference sample (without the addition of the extracts), their irritant potential, effect on skin hydration and pH, as well as transepidermal water loss from the epidermis (TEWL), were determined. 

## 2. Results and Discussion

### 2.1. Determination of Biologically Active Compounds

#### 2.1.1. Determination of Bioactive Compounds by HPLC-ESI/TOF

**Figure 1:**
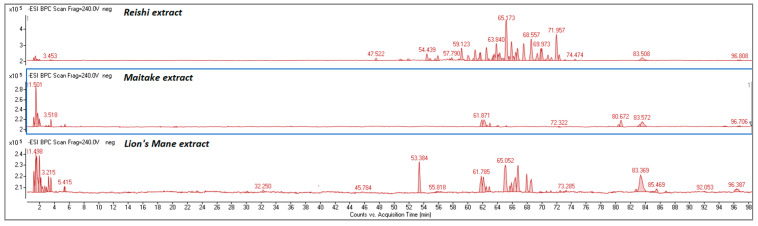
Representative BIC chromatogram for Reishi, Maitake and Lion’s Mane extracts.

The HPLC-ESI/TOF method was employed to investigate the chemical profiles of the Lion’s Mane, Reishi, and Maitake extracts. As can be seen in Figure 1, the extracts showed diverse phytochemical characteristics. The chromatogram of the Lion’s Mane extract shows many peaks at retention times from 0 to 4 min, which correspond to highly polar components; moreover, there are many peaks in the range of 53–70 min with one characteristic peak at 53.4 min (not identified, *m*/*z* 526.21). In the case of the Reishi extract, many peaks can be observed at retention times from 54 to 72 min. In turn, in the case of the Maitake extract, the number of registered peaks aresignificantly lower; the majority of peaks can beobserved in the region 0–4 min and some minor peaks are visible at 61–65 min. These observations demonstrate considerable differences in the composition of each extract.

Molecular weight, elution order and mass spectra were taken from literature data. Detailed results are shown in Table 1, Table 2 and Table 3. In this study, chemical compounds were identified in Lion’s Mane extract, including organic acids, aromatic compounds (hericerins) and diterpenoids (erinacines) (Table 1). Most of the compounds in Lion’s Mane exhibit good biological activity. Erinacines are able topromote the synthesis of nerve growth factors, thus have neuroprotective properties [35,36]. Phenolics compounds, as well as terpenoid lactones such as hericenone C, hericenone D, hericenone E, and hericenone H, can also promote the synthesis of nerve growth factors [37,38,39]. The anticancer properties of some of these compounds are also mentioned [40]. Table 2 shows the structure of the triterpenoids identified in the Reishi extract, most of which were ganoderic and lucidenic acids. The triterpenoids were extracted for HPLC-MS analysis at negative mode, which gave [M-H]- and [2 M-H]-ions. Furthermore, all tested mushroom extracts contained organic acids such as malic, fumaric, citric, pyroglutamic and (15z)-9,12,13-Trihydroxy-15-octadecenoic acid. 

#### 2.1.2. Total Phenolic Compounds and Flavonoids Content

Phenolic compounds and flavonoids are bioactive substances known for their antioxidant properties. In order to determine total phenolic content in water extracts of the mushrooms, the spectrophotometric method with the use of the Folin&Ciocalteu reagent was used. In order to determine the total flavonoids content, a spectrophotometric method with the use of the aluminum nitrate nonahydrate, was carried out. 

The obtained results are presented in Table 4. For all investigated mushroom extracts, the total content of phenolic compounds was higher than the total content of flavonoids. The highest content of phenolic compounds was found in Maitake (183.75 ± 0.21 µg GAE/g DW) which was about 13 times higher than the lowest content, which was obtained by Reishi (13.23 ± 0.07 µg GAE/g DW). The situation was similar in the case of the content of flavonoids. The highest value determined was for Maitake extract (38.38 ± 0.07 µg QE/g DW) and the lowest for Reishiextract (3.43 ± 0.03 µg QE/g DW). Although no polyphenolic and flavonoid compounds were found in the chromatographic analysis, the obtained results can be explained by the presence of non-phenolic components able to react with the aforementioned reagents. Reducing sugars are an example of such constituents, so total carbohydrates were determined in our study. The determined carbohydrate contents were 0.121 ± 0.011 mg/mL, 0.054 ± 0.003 mg/mL, and 0.015 ± 0.002 mg/mL for Maitake, Lion’s Mane, and Reishi, respectively. It can, therefore, be assumed that carbohydrates wereresponsible for the positive reaction with the Folin&Ciocalteu reagent.

### 2.2. Determination of Antioxidant Properties

**Figure 2:**
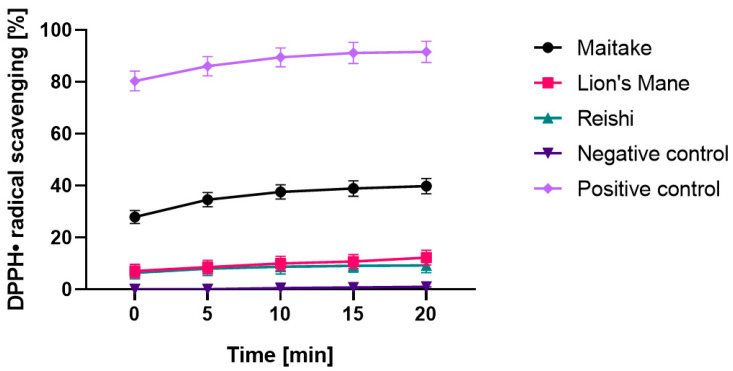
Kinetics of the absorbance changes in DPPH^•^ solutions in the presence of water extract of Maitake, Lion’s Mane and Reishi. Values are the mean of three replicate determinations Negative control: ethanolsolution (1000 µg/mL). Positive control: ascorbic acid solution (1000 µg/mL).

In order to determine the antioxidant properties of the tested extracts, a DPPH radical scavenging assay was performed. The obtained results, for the samples in a concentration of 1000 µg/mL, are presented in Figure 2. The most effective scavenging of the DPPH radical was noted for the Maitake extract, which reached the highest value after 20 min of incubation (39.84%). The Reishiextract obtained the least efficient scavenging of the DPPH radical and its value did not change during the 20 min of the measurement. At the beginning of the measurement, it was 6.47%, while after 20 min it was only 9.29%. The situation wassimilar in the case of the Lion’s Mane extract. At the beginning of the measurement, it was 7.11%, and after 20 min it was 12.29%.

**Figure 3:**
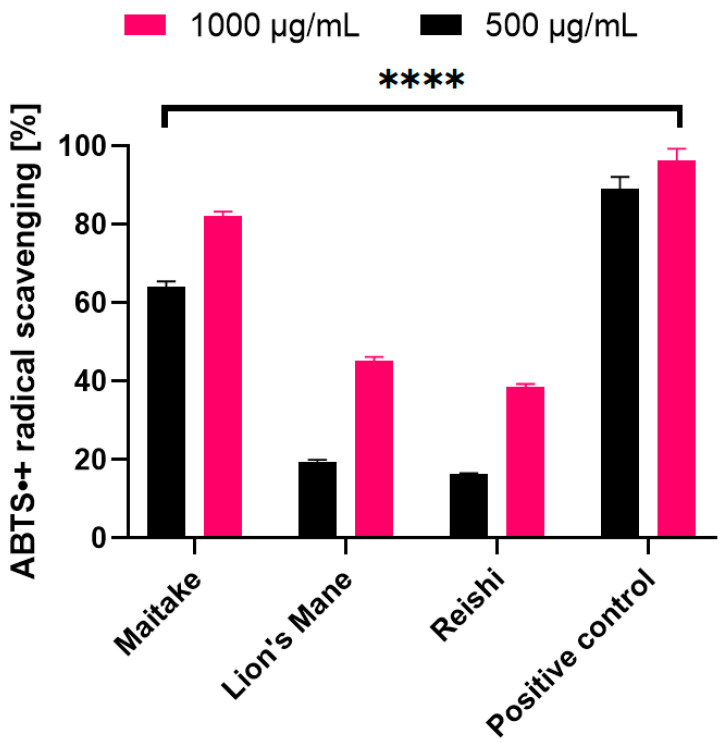
ABTS scavenging by extracts of Maitake, Lion’s Mane and Reishi. Data are the mean ± SD of three independent experiments, each of which consisted of three replicates per treatment group, **** *p* < 0.0001 versus the control. Negative control: ethanol solution (1000 µg/mL) (not shown). Positive control: ascorbic acid solution (500 and 1000 µg/mL).

**Figure 4:**
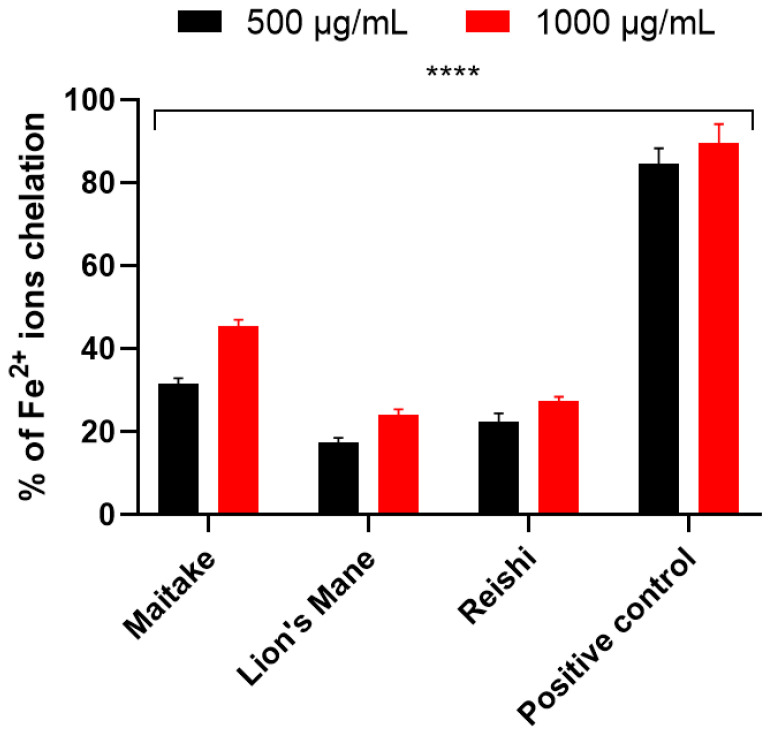
Chelation of Fe^2+^ by extracts of Maitake, Lion’s Mane and Reishi. Data are the mean ± SD of three independent experiments, each of which consisted of three replicates per treatment group, **** *p* < 0.0001 versus the control. Negative control: ethanol solution (1000 µg/mL) (not shown). Positive control: ascorbic acid solution (500 and 1000 µg/mL).

The second test used to determine the antioxidant properties of the examined extracts was ABTS^•^ scavengingassay. The obtained results, for the samples in concentrations of 500 and 1000 µg/mL, are presented on the Figure 3. For all extracts, the highest values of ABTS^•^ radical scavenging wereobtained in the concentration of 1000 µg/mL. The most effective scavenging was noted for Maitake (82.12%), and the least for Reishi (38.50%). The situation wassimilar in the case of the 500 µg/mLconcentration. The highest value was obtained for Maitake (64.19%) and the lowest for Reishi (16.22%). Due to the fact that Fe^2+^ ions are involved in the formation of reactive oxygen species, the next test evaluating the antioxidant properties was the test assessing the ability to chelate these ions (Figure 4). Results were obtained for extract samples also at concentrations of 500 and 1000 µg/mL. As in previous tests, the highest chelating capacity for Fe^2+^ ions was recorded for the Maitake extract (31.67%) in the concentration of 1000 µg/mL. The reducing activity of the tested Maitake, Lion’s Mane and Reishi extracts was also measured by the FRAP method (Table 5). The results of the experiments were expressed as μmol of Trolox equivalent/g of dry weight of individual mushrooms. As for the other tests assessing antioxidant activity, Maitake extract also showed the most favorable properties obtaining 21.1 ± 3.2 μmol Trolox/g dry weight.

Both for the extracts and for the prepared cosmetics containing the analyzed mushrooms, a test was carried out to detect the ability of the compounds to generate intracellular production of reactive oxygen species. The test was performed using fluorogenic H_2_DCFDA dye, which is oxidized inside cells into highly fluorescent 20,70-dichlorofluorescein in the presence of ROS. 

**Figure 5:**
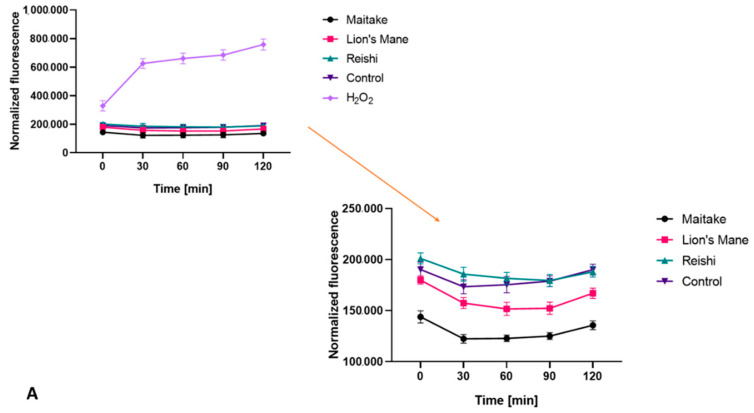

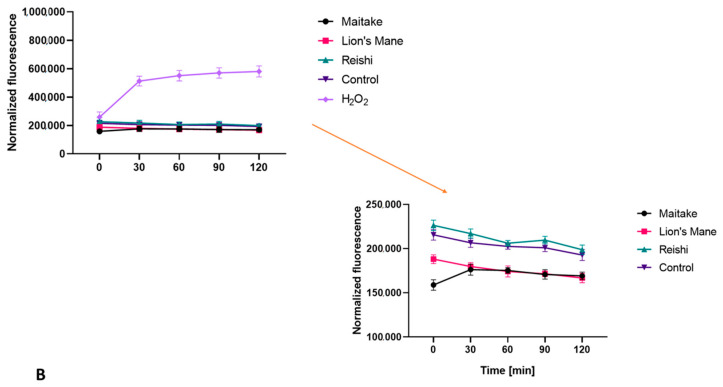
The effect of mushroom extracts at a concentration of 500 µg/mL on the 20,70-dichlorofluorescein (DCF) fluorescence in BJ (**A**), and HaCaT (**B**), cells. Data are the mean ± SD of three independent experiments.

As shown in Figure 5, all tested extracts did not increase the amount of ROS in fibroblasts. Reishi showed values similar to the control (cells not treated with the extracts), while Maitake and Lion’s Mane achieved values lower than the control, which means that the presence of these extracts lowers ROS levels in the cells. In this respect, the Maitake extract wasthe most effective, compared with the Reishi and Lion’s Mane extracts. The situation wassimilar with keratinocytes; only Reishi extract obtained a higher fluorescencevalue than the control. The rest of the extracts did not increase the intracellular production of ROS. 

**Figure 6:**
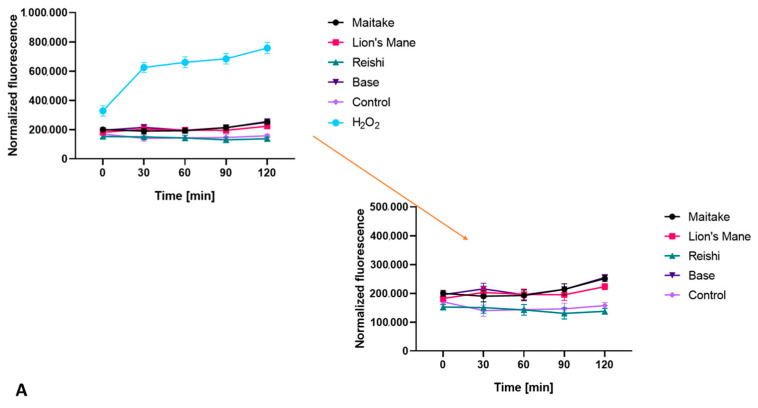

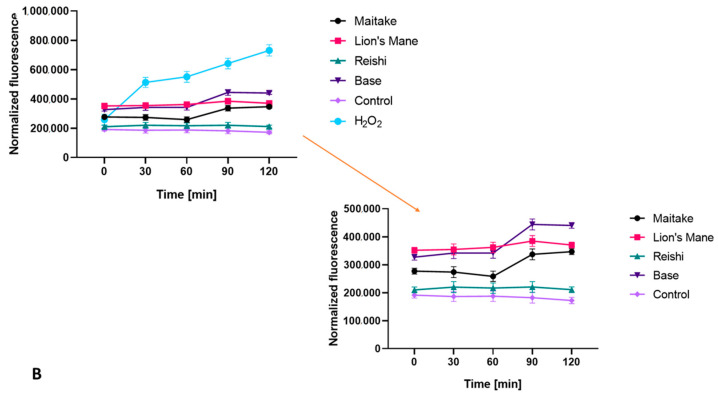
The effect of washing gels at a concentration of 0.01% on the 20,70-dichlorofluorescein (DCF) fluorescence in BJ (**A**), and HaCaT (**B**), cells. Data are the mean ± SD of three independent experiments.

A test was also carried out on washing gels containing extracts from the studied mushrooms, the results of which are presented in Figure 6. In the case of fibroblasts, all washing gels containing mushrooms extracts achieved higher fluorescence values than the control, but lower than cells treated with only base. In the case of keratinocytes, the situation wassimilar. The Maitake, Reishi and Lion’s Mane washing gels obtained a fluorescence value higher than the control, but lower than the base, which may suggest that the presence of the mushroom extract reduces the harmful effects of the compounds present in the cosmetic base, both on fibroblasts and keratinocytes. 

When analyzing the obtained results, it can be concluded that the tested mushrooms showed antioxidant activity. This is caused by the presence of compounds such as malic acid, fumaric acid, citric acid, hericenone, erinacine, ganoderic acid and lucidenic acid, which have the ability to protect cells against the harmful effects of free radicals [47,48,49,50,51,52,53,54,55,56]. Maitake extract showed the best antioxidant properties.

Wang and Xu investigated the antioxidant properties of various mushroom species, including Lion’s Mane and Maitake. They compared, among other, total phenolic content in acetone, ethanol, water, and hot water extracts. For both Lion’s Mane and Maitake, the highest phenolics were found in the aqueous extracts, namely, 3.08 mg GAE/g (Lion’s Mane) and 3.78 mg GAE/g (Maitake). Moreover, DPPH free radical scavenging capacities were determined, which proved to be better for Lion’s Mane (2.85 µmole TE/g) than Maitake (1.75 µmole TE/g) [57]. Yeh et al. tested two Maitake strains, for which the phenol content in the water extracts was 39.78 mg/g (T1 strain) and 38.96 m/g (T2 strain). The researchers also determined the content of flavonoids, which was lower: 1.09 mg/g for the T1 strain, and 0.52 mg/g for the T2 strain. The presence of ascorbic acid, which also exhibits antioxidant properties, was also confirmed in these extracts. The ability of the extracts to scavenge DPPH radicals was also tested; at a concentration of 20 mg/mL for cold water extracts of Maitake T1 and T2, it was 50.62% and 59.58%, respectively. Much higher results were recorded for ethanolic extracts: 99.19% (T1) and 84.36% (T2) at 20 mg/mL [58]. On the other hand, in the work of Rahman et al., the antioxidant properties of two Reishi strains were compared. Total polyphenol content for Ganoderma lucidum-5 was 33.30 mg/100g, and for Ganoderma lucidum-7 was 43.49 mg/100g. Total flavonoid content was 34.09 mg/100g (Ganoderma lucidum-5) and 38.08 mg/100g (Ganoderma lucidum-7). The ability to scavenge the DPPH radicals by strains Ganoderma lucidum-5 and Ganoderma lucidum-7, was 24.27% and 23.66%, respectively [59]. 

### 2.3. Cytotoxicity Assessment

**Figure 7:**
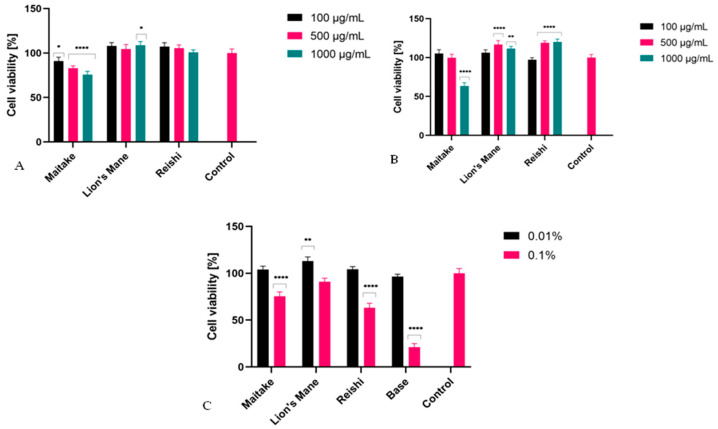
The reduction in resazurin after 24 h (for extract) and 1 h (for washing gels) exposure to the Maitake, Lion’s Mane and Reishi in cultured (**A**) fibroblasts (treated with extracts), (**B**) keratinocytes (treated with extracts), and (**C**) keratinocytes (treated with washing gels). Data are the mean ± SD of three independent experiments, each of which consisted of three replicates per treatment group, **** *p* < 0.0001, ** *p* < 0.005, * *p*< 0.8 versus the control (100%).

In assessing the potential use of extracts from various natural raw materials, including fungi, it is very important to assess their cytotoxicity to skin cells. Hence, as part of this study, the impact of the three prepared extracts, and gels containing these extracts, on the viability of fibroblasts and keratinocytes was assessed. The first of the tests to assess the metabolic activity of the studied cells was alamarBlueassay (AB). As shown in Figure 6, Lion’s Mane and Reishi extracts at all tested concentrations showed no toxic effects on keratinocytes (HaCaT) and fibroblasts (BJ cells). Maitake extract at the concentration of 100 µg/mL was not cytotoxic for BJ and HaCaT cells, but with the increase in concentration the cytotoxicity increased, and at the highest concentration (1000 µg/mL) the viability of these cells decreased below 60%. The developed gels in a concentration of 0.01%, containing extracts from the tested fungi, did not show any cytotoxicity. However, in the case of their higher concentration (0.1%), they showed cytotoxicity towards keratinocytes, depending on the type of fungus used. It should be noted that compared with the gels base, the addition of the extract significantly reduced their cytotoxicity, which indicates an increase in their safety of application to skin. The positive effect was observed to the greatest extent in the case of Lion’s Mane, for which at a concentration of 0.1%, an increase in keratinocyte viability by 70% compared with the gel base was observed. The cytotoxic effect of the gel base itself is probably related to the activity of surfactants present in the cosmetic base, the cytotoxic effect of which has been proven in numerous studies [60,61]. Based on the results presented in Figure 7C, it can be concluded that the presence of fungal extracts in the developed washing preparations minimizes the unfavorable effect of surfactants, and thus increases the proliferation and viability of cells.

**Figure 8:**
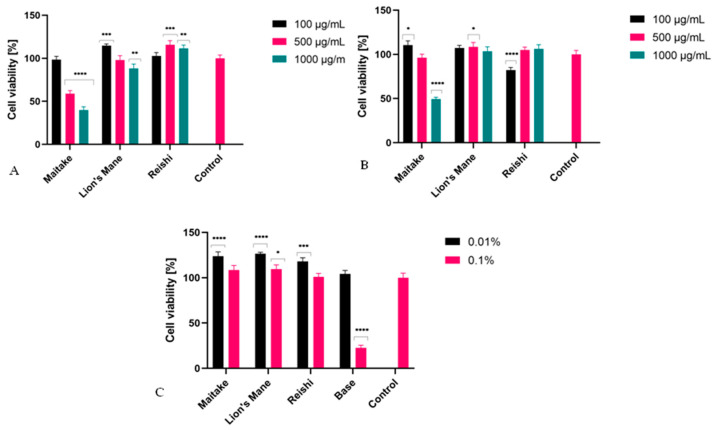
The effect of Maitake, Lion’s Mane and Reishi on Neutral Red dye uptake in cultured (**A**) fibroblasts (treated with extracts), (**B**) keratinocytes (treated with extracts), and (**C**) keratinocytes (treated with washing gels). The exposure time of cells to individual extracts was 24 h, and to washing gels, 1 h. Data are the mean ± SD of three independent experiments, each of which consisted of three replicates per treatment group, **** *p*< 0.0001, *** *p*< 0.0005, ** *p*< 0.005, * *p*< 0.05 versus the control (100%).

The evaluation of cytotoxicity was also carried out using the Neutral Red uptake assay (NR), and the results are shown in Figure 8. The obtained results indicate that Lion’s Mane and Reishi extracts at all tested concentrations showed no cytotoxicity to both BJ and HaCaT cells. Moreover, treating keratinocytes with Reishi extracts increased the viability of these cells. On the other hand, Maitake extract showed cytotoxicity to BJ cells at all tested concentrations, and this effect was stronger with increasing concentration. In the case of HaCaT cells, this extract showed no cytotoxicity at the concentrations of 100 µg/mL and 500 µg/mL, but it inhibited the viability of these cells at the concentration of 1000 µg/mL. In the case of the developed gels containing extracts of the tested mushrooms, none showed cytotoxicity. At a concentration of 0.01%, all showed the ability to increase the proliferation of HaCaT cells by about 20% compared with the control (cells not treated with gels). Similar to the AB test, the studies showed that the gel base at a concentration of 0.01% did not show cytotoxicity, while when 0.1% concentration wasused, the cytotoxicity wassignificant and reduced the keratinocyte viability to just over 20%. On the other hand, the addition of extracts from all three tested fungi to the gel formula eliminated the cytotoxicity of the tested washing preparations, which indicates the legitimacy of including these extracts in the formulathedeveloped gels.

Research on skin cell lines is an important element that allows assessment of the safety of the obtained extracts, and to predict their possible effects in the next stages of research, such as in vivo tests or clinical trials. The results obtained in this study, indicating the lack of cytotoxicity of the tested extracts used in a given concentration range and the possibility of reducing the negative effects of various cosmetic ingredients, allow the estimate that these raw materials can be more and more willingly included in the formulations of a wide range of cosmetic preparations. In addition to the lack of a negative impact on the viability and proliferation of keratinocytes and fibroblasts, scientific works indicate that these extracts also exhibit multidirectional activity in in vitro conditions.

The available literature data do not contain any reports on the cytotoxicity of Maitake extracts to skin cells. There are reports mainly describing antitumor, immunomodulating and antioxidant properties of this fungus, but the influence of this type of extract on the viability of keratinocytes and fibroblasts has not been assessed [62,63,64]. Contrary to the extracts of Lion’s Mane and Reishi, the analyses carried out in this study showed that these extracts, in the higher concentrations used, exert a cytotoxic effect on skin cells in vitro, mainly fibroblasts. This may be the result of the action of compounds contained in these extracts, including alpha-hydroxy acids, which, despite their antioxidant activity, may exert an antiproliferative effect on HaCaT cells [65]. Scientific research shows that malic acid inhibits the proliferation of keratinocytes by inhibiting the progression of the cell cycle in the G0/G1 phase. Additionally, this acid can induce the expression of endoplasmic reticulum stress-related proteins such as GRP78, GADD153 and ATF6α [65]. Other studies carried out on human dermal fibroblasts (HDF) exposed to ultraviolet A radiation, however, indicate that exopolysaccharide isolated from this fungus has photoprotective potential. This polysaccharide has an inhibitory effect on the expression of human interstitial collagenase (matrix metalloproteinase, MMP-1), which may reduce skin photoaging by reducing the matrix degradation system associated with MMP-1 [66]. Thus, in order to use the potential of this fungus, it is necessary to select theconcentration of the extract that will show the desired biological activity with the simultaneous lack of cytotoxicity. This is important because, as shown in this work, the addition of Maitake extract may reduce the negative effect of cosmetic cleansing preparations on skin cells.

The effect of Lion’s Mane extract on keratinocytes and fibroblasts in vitro has not been described to date. Thus, in this study, for the first time, we demonstrated the lack of cytotoxic effect of the studied extract on these skin cells, and the possibility of stimulating their viability and proliferation. This may be the result of reducing the level of free radicals in these cells, which was also shown in this work. The inhibitory effect on the production of ROS was also indicated by Chang et al., who demonstrated that the extracts of this fungus trigger the expression of the antioxidant genes of heme oxidase-1 (HO-1), γ-glutamylcysteine synthetase (γ-GCLC), and affect the level of glutathione. The antioxidant activity demonstrated by these authors on human endothelial cells (EA.hy926) is associated with increased nuclear translocation and transcriptional activation of NF-E2 associated factor 2 (Nrf2) [67].

Due to the fact that Reishi is considered to be one of the strongest adaptogens found in nature and exhibits not only antioxidant but also anti-inflammatory, immunomodulating and anti-cancer properties, interest in it in the context of skin care and treatment of skin diseases is growing [68]. Abate et al. showed that Reishiextract inducesthe proliferation of keratinocytes and increases the expression of cyclic protein kinases, such as CDK2 and CDK6. Additionally, these authors indicated that keratinocytes treated with Reishiextract show an increased migration rate and an increase in activation of tissue remodeling factors such as matrix metalloproteinases 2 (MMP2) and 9 (and MMP-9). Moreover, this extract, through its antioxidant activity, protects keratinocytes against H_2_O_2_-induced cytotoxicity. These studies indicate the legitimacy of the cosmetic use of this fungus due to the possibility of accelerating wound healing processes, protecting cells against oxidative stress and intensified re-epithelialization [69]. Kim et al. indicated the inhibitory effect of Reishi extract on the activity of tyrosinase and melanin biosynthesis in B16F10 melanoma cells. They also demonstrated the possibility of inhibiting the expression of tyrosinase-related protein 1 (TRP-1), TRP-2, as well as microphthalmia-related transcription factor (MITF), thus reducing the production of melanin. This extract also influences the mitogen-activated kinase (MAPK) cascade and cyclic adenosine monophosphate (cAMP)-dependent signaling pathway, which has a significant effect on the melanogenesis of B16F10 melanoma cells [20]. Hu et al., on the other hand, indicated that the polysaccharides contained in Reishi increase the viability of fibroblasts and the ability to migrate these cells. Moreover, these polysaccharides increase the expression of β-catenin, CICP and TGF-β1 in fibroblasts in vitro. Additionally, in vivo studies in mice indicated that these compounds significantly improved healing rates and wound healing time. This is likely the result of activation of the Wnt/β-catenin signaling pathway and elevated levels of TGF-β1 [1]. 

To sum up, the lack of cytotoxic effect of Lion’s Mane and Reishi extracts on skin cells, and a reduction in the cytotoxicity of the base of cleansing preparations by extracts from all three tested mushrooms, indicate that they can be perceived as valuable cosmetic raw materials with a broad spectrum of activity.

### 2.4. Transepidermal Water Loss (TEWL), Skin Hydration and Skin pH Measurements

**Figure 9:**
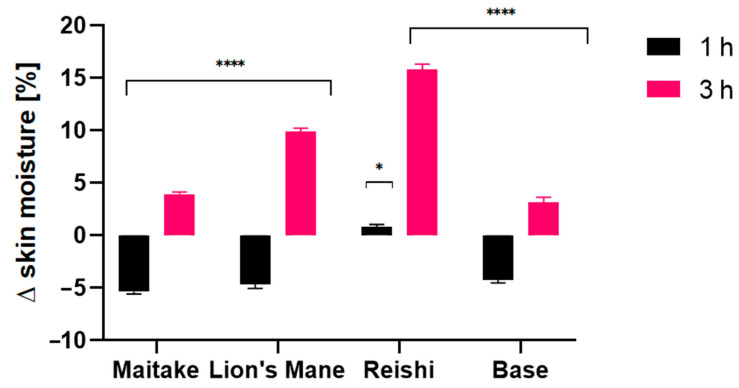
The influence of model gels with Maitake, Lion’s Mane and Reishi extracts, on skin hydration. Data are the mean ± SD of three independent measurements. **** *p* < 0.0001, * *p* < 0.015 versus the control.

**Figure 10:**
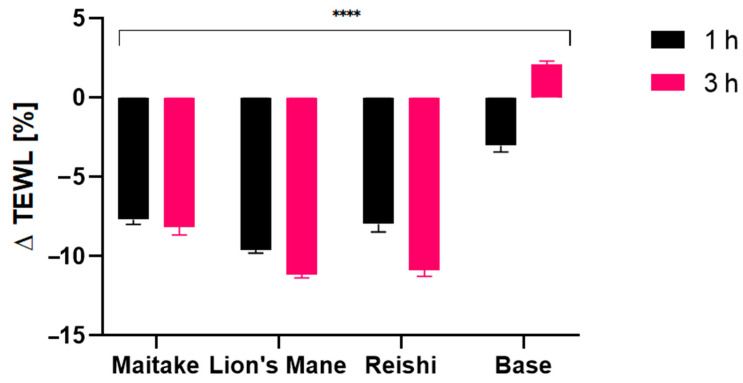
The influence of model gels with Maitake, Lion’s Mane and Reishi extracts, on transepidermal water loss (TEWL). Data are the mean ± SD of three independent measurements. **** *p* < 0.0001 versus the control.

Due to the content of hydroxyl groups in molecules, bioactive ingredients of the studied extracts have a positive effect on the condition of our skin. They mainly affect the retention of water in the skin, but also reduce the evaporation of water from the upper layer of the epidermis [70,71,72]. In addition, these extracts are characterized by several health-promoting properties and therefore can be successfully used as active ingredients in cosmetic products. The conducted research assessed the effect of preparations containing extracts on basic skin parameters, such as skin hydration, TEWL and pH. The tested samples were products containing 1% mushroom extract (Reishi, Lion’s Mane, Maitake) and the base sample, for conductinga comparison. The analyses were carried out at twotime intervals, 1 h and 3 h, after the application of the product. The analysis of the obtained results showed the positive effect of the contained extract on the condition of the skin. The results are presented in the Figure 8, Figure 9 and Figure 10.

Analysis of skin moisture showed differences between examined model gels with extracts, which will be discussed one by one. For the gel containing Maitake extract, aminimal influence was observed. After 1 h, the effect was similar to the base, for which decreased moisture was recorded, but registered values were around 20% lower when compared with the base. After 3 h it turned into a 20% relative increase with reference to the base level. In the case of Lion’s Mane, the initial effect measured after 1 h was also negative, showing around 10% decrease in moisture compared with the base, but after 3 h the result turned into a significant 200% increase in moisture. The best results were noted for the Reishi extract, where, even after the first 1 h, a slight increase in moisture was observed, bearing in mind that for the base a noticeable decrease was observed. After 3 h, high values were observed, as the moisture was 350% higher when compared with the base. From that perspective, Reishi showed the most preferable properties, with better results, compared with the rest of the examined extracts.

Analysis of the TEWL showed a positive effect of each of the model gels with mushroom extracts, however there were still some differences between them which are worth discussing. First, values observed after 1 h were all preferable, showing values around −7.5% and −8% for Maitake and Reishi, respectively, and even better valuearound −9.5% for Lion’s Mane. Values measured after 3 h showed, in case of Maitake extract, the TEWL value stayed at almost the same level of −8%, but in the case of Lion’s Mane and Reishi extracts even better values were observed, equaling −11% for both. In conclusion, the Lion’s Mane extract exhibited the most preferable properties, slightly ahead of the Reishi extract.

**Figure 11:**
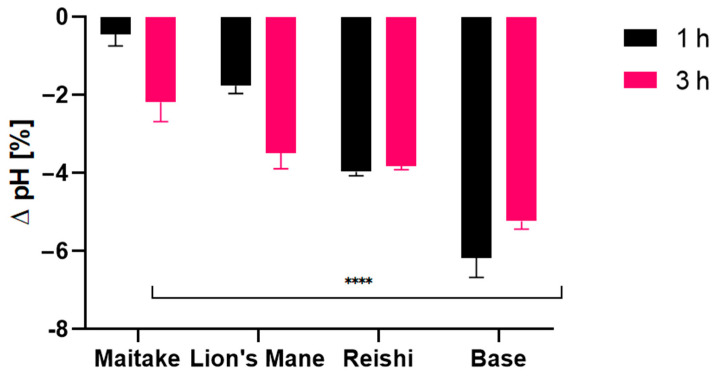
The influence of model gels with Maitake, Lion’s Mane and Reishi extracts on skin pH. Data are the mean ± SD of three independent measurements. **** *p* < 0.0001 versus the control.

Analysis of the pH showed noticeable differences between model gels with mushroom extracts (Figure 11). Zero level means no change in the skin pH in relation to the control field, i.e., the physiological pH of the test volunteers, therefore, the most favorable values would beclose to zero. First, values observed for the base werethe most distant from zero level, being the least preferable. Values observed for the Maitake extract appeared to be the best among those compared, showing differences of −0.5 and −2, respectively, after 1 h and 3 h. It is worth emphasizing that the deviation observed after 1h wasvery close to the perfect natural skin pH.NextwastheLion’s Mane extract, for which differences of −2 and −3.5,after 1 h and 3 h, respectively, wereobserved. The worst results were measured for the Reishi extract, where the deviation from the optimal pH was close to −4 after both 1 h and 3 h. From that perspective, the Maitake extract results werethe most preferable.

### 2.5. Irritant Potential of Model Body Wash Gels

**Figure 12:**
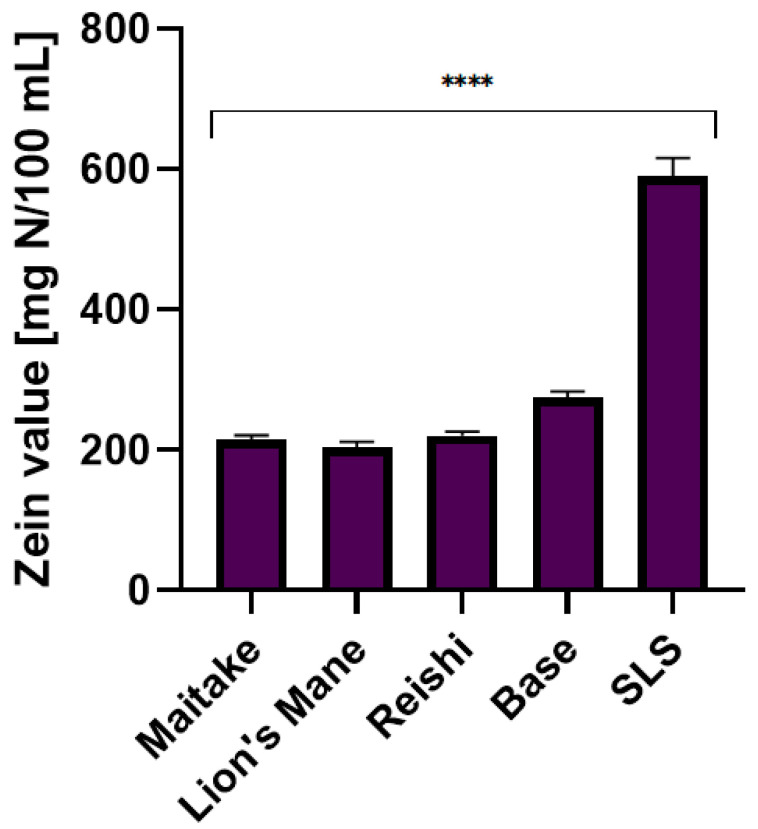
Irritant potential of model washing gels with Maitake, Lion’s Mane and Reishi extracts. Data are the mean ± SD of three independent measurements. **** *p* < 0.0001 versus the control. Positive control: sodium lauryl sulfate (SLS) solution (1%).

The lack of adverse effects of washing cosmetics on skin is one of the most important requirements for this group of products. The measurement results of the irritating potential of the analyzed washing gels are presented in Figure 12.

The method used to determine the irritation potential of the model washing gels was the determination of the zein number. Due to the fact that the irritant potential of washing cosmetics is caused by interactions of surfactants with proteins that build the stratum corneum layer, this method closely imitates the strength of the interactions of surfactants used in the formulation of the model washing gels, with skin proteins. The irritating potential generated by surfactants results from the ability of these substances to denature epidermal proteins and then wash them out of the skin. Contact of the analyzed samples with zein, a protein with a structure similar to the main proteins in skin, may indicate their protein-denaturing capacity, which is a measure of irritating potential. 

Obtained results showed that the addition of analyzed mushroom extracts to the base model washing gel reduced the irritant potential (zein number). The base sample (without the addition of the extracts) was characterized by a zein number at the level of 270 mg N/100 mL and, according to the accepted classification, was classified as moderately irritant for the skin. Samples containing Maitake and Reishi extracts had lower irritant potential and their zein valueswere about 20% lower in relation to the base sample (about 220 mg N/100 mL). The lowest irritant potential was observed for the sample that contained Lion’s Mane extract. The zein value of this sample was about 25% lower than the base sample. The obtained results indicate that samples with the addition of the analyzed extracts can be classified on the border of non-irritating and slightly irritating. 

The anionic surfactant, sodium coco sulfate, was used in the formulation of the analyzed model washing gels as the main washing agent. As mentioned in the introduction, anionic surfactants have the strongest irritant potential due to the fact that they interact with skin proteins through strong ionic bonds, and their denaturing potential is significantly higher than for nonionic and amphoteric surfactants. The lower irritant potential observed for the samples containing mushroom extracts wasmost probably caused by carbohydrates and proteins, that are the main ingredients of mushrooms’ dry weight, and are extracted, in the extraction process, by polar solvents. These substances may be incorporated into surfactant’s micelles, causing anincrease in their stability and size. Formation of mixed micelles that contain both surfactants and high molecular weight carbohydrates and proteins limits the number of free surfactants in the form of monomers that are released to the bulk phase, and leads to a lowering of the irritant potential of the product [23,25,26,27].

## 3. Materials and Methods

### 3.1. Chemicals

2′,7′-dichlorodihydrofluorescein diacetate (H_2_DCFDA, Sigma-Aldrich, Poznan, Poland), acetic acid (CH_3_COOH, ≥99%, Sigma-Aldrich, Poznan, Poland), acetonitrile (CH_3_CN, ≥99.9%, Sigma-Aldrich, Poznan, Poland), aluminum nitrate nonahydrate (Al(NO_3_)_3_·9H_2_O, Sigma-Aldrich, Poznan, Poland), antibiotics (Penicillin–Streptomycin, Life Technologies, Bleiswijk, The Netherlands), DMEM (Dulbecco’s modification of Eagle’s medium, Biological Industries, Beit Haemek, Israel), ethyl alcohol (ethanol, C_2_H_5_OH, 96%, Sigma-Aldrich, Poznan, Poland), FBS (fetal bovine serum, Biological Industries, Genos, Lodz, Poland), Folin &Ciocalteu′s phenol reagent (Sigma-Aldrich, Poznan, Poland), formic acid (HCOOH, Merck Life Science, Poznan, Poland), gallic acid (C_7_H_6_O_5_, Sigma-Aldrich, Poznan, Poland), methanol (CH_3_OH, ≥99.9%, Sigma-Aldrich, Poznan, Poland), Neutral Red solution (NR, 0.33%, Sigma-Aldrich, Poznan, Poland), phosphate buffered saline (PBS, pH 7.00 ± 0.05, ChemPur, Piekary Slaskie, Poland), potassium persulfate (99.99%, K_2_S_2_O_8_, Sigma-Aldrich, Poznan, Poland), resazurin sodium salt (RES, Sigma-Aldrich, Poznan, Poland), sodium carbonate (Na_2_CO_3_, ≥99.5%, Sigma-Aldrich, Poznan, Poland), Sodium Pyruvate Solution (100 mM, Genos, Lodz, Poland), trypsin-EDTA solution (Sigma-Aldrich, Poznan, Poland), Quercetin hydrate (≥95%, Sigma-Aldrich, Poznan, Poland), were used as received.

### 3.2. Preparation of Extracts

The material used in the research was dry, powdered mushrooms: Maitake, Lion’s Mane and Reishi, purchased from the Polish certified dealer, MagicznyOgród. To prepare the extracts, 5 g of powdered mushroom was weighed into a beaker. and 100 mL of water was added. Afterwards, the beakers were placed in an ultrasonic bath (Digital Ultrasonic Cleaner) for 5 min. After this time, the obtained extracts were filtered through Whatman No. 1 filters. After filtration, the extracts were evaporated in a concentrator at a temperature of 40 °C under reduced pressure. From the obtained dry extracts, stocks with a concentration of 100 mg/mL were prepared and stored at 4 °C for further analysis.

### 3.3. Determination of Biologically Active Compounds

#### 3.3.1. Determination of Bioactive Compounds by HPLC-ESI/TOF

Extracts were analyzed using ultra-high performance liquid chromatograph (UHPLC) with an ESI/TOF detector (Agilent Technologies, Santa Clara, CA, USA). The separation was carried out on an RP18 reversed-phase column Titan (10 cm × 2.1 mm i.d., 1.9 µm particle size) (Supelco, Sigma-Aldrich, Burlington, MA, USA) using a mixture of water with 0.05% of formic acid (solvent A) and acetonitrile with 0.05% of formic acid (solvent B) at a flow rate of 0.2 mL/min according to gradient as follows: 0–5 min from 100% A to 98% A (from 0% to 2% B), 5–50 min from 98% A to 75% A (from 2% to 25% B), 50–70 min from 75% A to 60% A (from 25% to 40% B), and 70–100 min from 60% A to 40% A (from 40% B to 60% B). Thermostat temperature was 30 °C. The ion source operating parameters were as follows: drying gas temperature 325 °C, drying gas flow 5 L min^−1^, nebulizer pressure 30 psi, capillary voltage 3500 V, fragmentator 240 V, and skimmer 65 V. Ions were acquired in the range of 100 to 1000 *m*/*z*.

#### 3.3.2. Determination of the Total Phenolic Content (TPC)

In order to determine the TPC of analyzed extracts, the spectrophotometric method involving the use of the Folin&Ciocalteu reagent, was carried out [70]. Gallic acid solution in a concentration range of 10–100 mg/mL was used as astandard. First, 1500 µL of 1:10 diluted Folin&Ciocalteu reagent was added to the test tubes containing 300 µL of the analyzed samples (in concentrations of 100, 500 and 1000 µg/mL) and incubated in adark room for 6 min. Then, 1200 µL of 7.5% sodium carbonate solution was added to the test tubes. Samples were mixed and incubated in the dark room for 1.5 h. After this time, the absorbance was measured at wavelength of λ = 740 nm. To calculate the total concentration of phenols in the analyzed samples, a gallic acid (GA) calibration curve (in the 10–100 mg/mL concentration range) was used. The measurements were made in triplicate and the results obtained were averaged. The TPC results are presented as µg of GA equivalents (GAE) per g of dry weight.

#### 3.3.3. Determination of the Total Flavonoids Content (TFC)

In order to determine the TFC of analyzed extracts, the spectrophotometric method involving the use of aluminum nitrate nonahydrate, was carried out [71]. Quercetin hydrate solution in a concentration range of 10–100 mg/mL was used as atandard. In the first step, 1200 µL of reaction mixture containing 80% C_2_H_5_OH, 10% Al(NO_3_)_3_·9H_2_O, and 1M C_2_H_3_KO_2_, was added to 300 µL of the analyzed samples (in concentrations of 100, 500 and 1000 µg/mL). Then, samples were mixed and incubated in a dark room for 40 min. After this time, the absorbance was measured at wavelength of λ = 415 nm. To calculate the total concentration of flavonoids in the analyzed samples, a quercetin (Q) calibration curve was used. The measurements were made in triplicate and the results obtained were averaged. The TFC results are presented as µg of Q equivalents (QE) per g of dry weight.

#### 3.3.4. Determination of Total Carbohydrate

The total carbohydrate content in the extracts was estimated using the phenol sulfuric acid method. An amount of 0.25 mL of sample was mixed with 1.25 mL of concentrated sulfuric acid (95% *v*/*v*) and 0.25 mL of phenol (5% *v*/*v*). The mixture was heated at 100 °C for 5 min, cooled to room temperature and absorbance was measured at 490 nm. The results were calculated using the glucose calibration curve [72].

### 3.4. Determination of Antioxidant Properties

#### 3.4.1. DPPH Radical Scavenging Assay

The ability to scavenge free radicals was determined using the method described by Brand-Williams et al. [73], which is based on using the 1,1-diphenyl-2-picrylhydrazyl (DPPH) radical. In the first step, 100 µL of water solutions of analyzed extracts at concentration of 1000 µg/mL were transferred to a 96-well plate. Then, 100 µL of ethanol solution of DPPH was added to the samples and mixed. An ethanol solution was used as a negative control, and an ascorbic acid solution as a positive control, at a concentration of 1000 µg/mL. The absorbance was measured at wavelength of 517 nm, every 5 min for 20 min, using a UV-VIS Filter Max spectrophotometer (Thermo Fisher Scientific, Waltham, MA, USA). Measurements were carried out in triplicate for each extract sample. The antioxidant capacity was expressed as a percentage of DPPH inhibition using Equation (1):

(1) $$\%\mathrm{DPPH}\;\mathrm{scavenging}=\frac{\mathrm{Abs}\;\mathrm{control}-\mathrm{Abs}\;\mathrm{sample}}{\mathrm{Abs}\;\mathrm{control}}\times 100$$

where: Abs sample—absorbance of the sample; Abs control—absorbance of the control sample. 

#### 3.4.2. ABTS^•^ Scavenging Assay

The second method for determining the antioxidant properties of mushroom water extracts was by ABTS^•^ scavenging assay, described by Gaweł-Beben et al. [74]. First, a 7 mM aqueous ABTS solution and 2.4 mM potassium persulfate were prepared. Then, ABTS and potassium persulfate were mixed in equal proportions and left at room temperature, in darkness for 14 h. After this time, the solution was diluted in methanol to an absorbance at the level of about 1.0 (λ = 734 nm). In the next step, 1 mL of analyzed extract in a concentration of 1000 µg/mL was mixed with 1 mL of ABTS. An ethanol solution was used as a negative control, and an ascorbic acid solution as a positive control, at a concentration of 500 and 1000 µg/mL. Distilled water was added to the blank instead of the extract. The absorbance was measured at wavelength of λ = 734 nm using a UV/VIS spectrophotometer Aquamate Helion (Thermo Fisher Scientific, Waltham, MA, USA). The ABTS^•^ scavenging was calculated from Equation (2):

(2) $$\%\;\mathrm{of}\;{\mathrm{ABTS}}^{•}+\mathrm{scavenging}=1-\frac{\mathrm{As}}{\mathrm{Ac}}\times 100$$

where: As—absorbance of the sample; Ac—absorbance of the control sample.

#### 3.4.3. Fe^2+^ Chelation Assay 

Chelation of iron (II) ions by Maitake, Lion’s Mane and Reishi extracts was measured according to the methodology described by Gaweł-Bęben et al. [74] with slight modifications. In the first step, 0.5 mL of each extract (with a concentration of 500 and 1000 µg/mL) was mixed with 3.7 mL of distilled water, 0.1 mL of FeCl_2_ (1 mM) and 0.2 mL of ferrosine (5 mM). An ethanol solution was used as a negative control, and an ascorbic acid solution as a positive control, at a concentration of 500 and 1000 µg/mL.Then, the reaction mixture was thoroughly mixed and incubated at room temperature for 10 min. The absorbance of the tested samples was measured at wavelength of λ = 562 nm using a UV/VIS spectrophotometer Aquamate Helion (Thermo Fisher Scientific, Waltham, MA, USA). Each sample was analyzed in triplicate. The chelating activity of the analyzed mushroom extracts was calculated as the percentage inhibition of the ferrosine-Fe^2+^ complex formation from the formula:

% Fe^2+^ chelating activity = [1 − (As/Ac)] × 100%

where: As—sample absorbance; Ac—absorbance of the control sample.

#### 3.4.4. Ferric Reducing Antioxidant Power (FRAP) Assay

The reducing activity of the studied Maitake, Lion’s Mane and Reishi extracts was also measured on the basis of the FRAP method procedure described by Benzie and Strain, with minor modifications [75]. In the first step, fresh FRAP reagent was prepared. For this purpose, 25 mL of acetate buffer (CH_3_COOH:CH_3_COONa, 0.3 M, pH = 3.6) wasmixed with 25 mL of methanol (Alchem, Toruń, Poland). An amount of 5 mL of 2,4,6-tris (2-pyridyl)-s-triazine (10 mM) dissolved in HCl (0.04 M) and 5 mL of FeCl_3_·6H_2_O (0.02 M) were then added to the solution. In the next step, 225 µL of 50% methanol solution and 75 µL of the tested 1000 µg/mL mushroom extracts were added to 2.25 mL of FRAP reagent and mixed thoroughly. The mixture was incubated in a 37 °C water bath for 30 min. The absorbance was then measured at λ = 593 nm in a glass cuvette using an Aquamate Helion spectrophotometer (Thermo Fisher Scientific, Waltham, MA, USA). A standard curve was prepared using different Trolox concentrations. For each extract, measurements were made in triplicate. The results of the experiments were expressed as μmol of Trolox equivalent/g of dry weight of individual mushrooms.

#### 3.4.5. Detection of Intracellular Levels of Reactive Oxygen Species (ROS)

In order to determine the ability of the analyzed extracts and washing gels to generate the intracellular production of reactive oxygen species in skin cells, a fluorogenic H_2_DCFDA dye was used. This dye has the ability to penetrate inside the cell, where it is transformed into a non-fluorescent compound. If reactive oxygen species are present in the cell, this compound is then transformed into highly fluorescent DCF. To determine the intracellular level of ROS in HaCaTs and BJ, cells were seeded in 96-well plates and cultured in an incubator for 24 h. Next, DMEM medium was removed and replaced with 10 µM H_2_DCFDA (Sigma Aldrich, St. Louis, MO, USA) dissolved in serum-free DMEM medium. Cells were incubated for 45 min and then incubated with the extracts in concentrations of 100, 500, and 1000 µg/mL, and with samples of washing gels in concentrations of 0.1% and 0.01%. Cells treated with 1 mM hydrogen peroxide (H_2_O_2_) were used as positive controls. The control samples were cells untreated with the tested extracts. The fluorescence of DCF was measured every 30 min for 120 min using a FilterMax F5 microplate reader (Thermo Fisher Scientific) at a maximum excitation of 485 nm and emission spectra of 530 nm [76].

### 3.5. Cytotoxicity Analysis

#### 3.5.1. Cell Culture

In this research, two types of skin cells were used: fibroblasts (American Type Culture Collection Manassas, VA, USA) and keratinocytes (CLS Cell Lines Service GmbH, Eppelheim, Germany). Cells were grown in Dulbecco’s Modification of Eagle’s Medium (DMEM, Biological Industries, Cromwell, CO, USA) high glucose content (4.5 g/L), enriched with sodium pyruvate, L-glutamine, 10% fetal bovine serum (Gibco, Waltham, MA, USA) and 1% antibiotics (100 U/mL penicillin and 1000 µg/mL streptomycin, Gibco). Cells were grown in an incubator in a humidified atmosphere of 95% air and 5% carbon dioxide at 37 °C. After obtaining the required confluence, the medium was removed. Cells were washed with sterile phosphate buffered saline and then were detached from the bottom of the culture flasks with trypsin. Next, cells were placed in fresh medium, plated in 96-well plates and incubated for 24 h. After this time, cells were treated with mushroom extracts in concentrations of 100, 500 and 1000 µg/mL, and washing gels in concentrations of 0.01% and 0.1% containing mushrooms extracts, and incubated for another 24 h.

#### 3.5.2. AlamarBlue Assay

In order to evaluate the viability of BJ and HaCaT cells treated with analyzed extracts, the alamarBlueassay was performed. After incubation, analyzed samples were removed from the wells and then Resazurin solution (60 µM) was added. Plates were placed in an incubator for 2 h at 37 °C. Then, fluorescence was measured at wavelength of λ = 570 nm. Each sample was performed in three replications.

#### 3.5.3. Neutral Red Uptake Assay

In addition, the Neutral Red uptake assay was performed. After incubation, analyzed samples were removed from the wells and then Neutral Red dye (40 µg/mL) was added to the wells. Plates were placed in an incubator for 2 h at 37 °C, then the Neutral Red dye was removed, and the cells were washed with phosphate buffered saline. After this, phosphate buffered saline was removed and 150 µL of decolorizing buffer was added. The absorbance measurements were performed at wavelength of λ = 540 nm. Each sample was performed in three replications.

### 3.6. Transepidermal Water Loss (TEWL), Skin Hydration and Skin pH Measurements 

TEWL, skin pH and skin hydration measurements were conducted using a Tewameter TM 300 probe, Skin pH Meter PH950 and a Corneometer CM825 probe connected to a MPA adapter (Courage + Khazaka Electronic, Köln, Germany). The study was conducted on 10 volunteers, according to the procedure described by Nizioł-Łukaszewska et al. [77]. Five areas (2 cm × 2 cm in size) were marked on the forearm skin of volunteers. Then, 0.2 mL of 100 µg/mL solution of obtained gelswas applied to four fields (50 µL/cm^2^). One field (control field) was not treated with any sample. Sample solutions were gently spread over every part of the four fragments of the skin in the marked area, and after 20 min, dried with a paper towel. After 60 and 180 min, the hydration and TEWL measurements were taken. The final result was the arithmetic mean (from each volunteer) of five independent measurements (skin hydration and pH) and 20 measurements (TEWL). The final result was expressed as the change in skin hydration, TEWL and pH after the application of the analyzed gels relative to the control field to which the product was not applied.

### 3.7. Determination of Irritant Potential–Zein Value

Irritant potential of the products was measured using the zein test [18,78]. In a solution of surfactants, the zein protein denatures and then dissolves in the solution. This process simulates the behavior of surfactants towards skin proteins. Zein from corn, in the amount of 2 ± 0.05 g, was added to 40 mL of each of the samples. Sodium lauryl sulfate (1%) was used as a positive control. The solutions with zein were shaken in a shaker in a water bath (60 min at 35 °C). The solutions were filtered using Whatman No. 1 filters and then centrifuged at 5000 rpm for 10 min. The nitrogen content in the solutions was determined by Kjeldahl method. One milliliter of the filtrate was mineralized in sulfuric acid (98%) containing copper sulphate pentahydrate and potassium sulphate. After mineralization, the solution was transferred (with 50 mL of Milli-Q water) into the flask of the Wagner–Parnas apparatus. In the next step, 20 mL of sodium hydroxide (25 wt%) was added. The released ammonia was distilled with steam. Ammonia was bound by sulfuric acid (5 mL of 0.1 N H_2_SO_4_) in the receiver of the Wagner–Parnas apparatus. The unbound sulfuric acid was titrated with 0.1 N sodium hydroxide. Tashiro solution was used as an indicator. The zein number (ZN) was calculated using Equation (3):

(3) ZN=(10−V1)×100×0.7 [mg N/100 mL]

where V1 is the volume (mL) of sodium hydroxide used for titration of the sample. The final result was the arithmetic mean of five independent measurements.

### 3.8. Preparation of the Model Washing Gels

The final formulation of the analyzed model washing gels is presented in Table 6. Raw materials widely used in the cosmetics industry were used to prepare the samples.

We prepared 600 g of the washing gel according to the following procedure: water was weighed into the glass beaker and then heated to 45 °C. Raw materials, in the order given in the formulation, were added to the water and mixed (mechanic stirrer Chemland O20) until fully dissolved. Then, the obtained gel was cooled to room temperature and divided into 4 equal parts. One was the base model washing gel without the addition of the extract. Maitake, Reishi and Lion’s Mane extracts were added to the three remaining samples at a concentration of 1 wt% in each sample.

### 3.9. Statistical Analysis

Values of different parameters were expressed as the mean ±standard deviation (SD). Two-way analysis of variance (ANOVA) was performed at the *p*-value level of <0.05 to evaluate the significance of differences between values. Statistical analysis was performed using GraphPad Prism 8.0.1 (GraphPad Software, Inc., San Diego, CA, USA).

## 4. Conclusions

The paper presents the potential use of mushroom extracts in cosmetics. Three types of mushrooms were tested—*Grifolafrondosa* (Maitake), *Hericiumerinaceus* (Lion’s Mane) and *Ganoderma Lucidum* (Reishi)—as potential ingredients in cleansing cosmetics. The obtained extracts were characterized by moderate antioxidant properties, and the strongest properties were demonstrated by the Maitake extract. Reishi and Lion’s Mane extracts did not show cytotoxic activity, while Maitake extract in concentrations above 500 µg/mL may be toxic to skin cells, keratinocytes and fibroblasts. The introduction of extracts from the analyzed mushrooms to the recipes of model washing gels had a positive effect on their safety. It was shown that samples with the addition of the extract at the level of 0.1% were characterized by significantly lower toxicity to skin cells, compared with the sample without the addition of mushroom extract. Furthermore, addition of the analyzed extracts to the formulas of shower gels had a positive effect on skin hydration, TEWL and skin pH after their application. Compared with the base washing gel (without the addition of an extract), samples containing extracts in the recipe moisturized the skin more strongly and reduced TEWL. The most favorable properties were observed for a gel sample with the addition of Reishi extract. This study found that a very important property of mushroom extracts was their ability to reduce the irritating effect of washing gels; the analyzed extracts used in a concentration of 1% reduced the irritating effect of the product by up to 20%. The conducted research confirmed that the analyzed mushroom extracts contain a number of bioactive substances that have a positive effect on skin and may be a valuable active ingredient in cosmetic products.

## Figures and Tables

**Table 1:** Results of compound identification in Lion’s Mane extract using HPLC-ESI/TOF.

Rt (min.)	*m*/*z*-H	Error (ppm)	Molecular Formula	Compound	Identification
1.90	133.0142	−0.35	C_4_H_6_O_5_	Malic acid	[41]
1.96	479.1275	3.93	C_15_H_28_O_17_	Unknown	
2.09	115.0042	4.46	C_4_H_4_O_4_	Fumaric acid	[41]
2.15	523.0952	2.15	C_19_H_24_O_17_	Unknown	
3.32	128.0350	−2.45	C_5_H_7_NO_3_	Pyroglutamic acid	[41]
5.41	474.1567	−4.88	C_17_H_31_O_15_	Unknown	
32.25	209.0445	−4.98	C_10_H_10_O_5_	Unknown	
53.38	526.1752	0.26	C_17_H_35_O_18_	Unknown	
61.78	329.2340	1.98	C_18_H_34_O_5_	(15z)-9,12,13-Trihydroxy-15-octadecenoic acid	[41]
62.05	329.1389	−1.66	C_19_H_22_O_5_	Hericenone A	[42]
62.50	329.2331	−0.75	C_18_H_34_O_5_	Octadecenoic acid derivatives	[41]
62.90	329.2342	2.58	C_18_H_34_O_5_	Octadecenoic acid derivatives	[41]
65.05	431.2445	1.36	C_25_H_36_O_6_	Erinacine A	[43]
67.70	473.1812	−1.07	C_25_H_30_O_9_	Unknown	
66.39	473.1803	−2.97	C_25_H_30_O_9_	Unknown	
66.77	477.2868	2.14	C_27_H_42_O_7_	Erinacine D	[42]
67.65	432.2188	1.77	C_27_H_31_NO_4_	Hericenone B	[42]
68.57	473.1821	0.83	C_25_H_30_O_9_	Unknown	
83.37	265.1471	−3.02	C_12_H_26_O_4_S	Dodecyl sulfate	[41]
96.725	309.2790	−2.91	C_20_H_38_O_2_	Ethyl oleate	[41]

**Table 2:** Results of compound identification in Reishi extract using HPLC-ESI/TOF.

Rt (min.)	*m*/*z*-H	Error (ppm)	Molecular Formula	Compound	Identification
**1.95**	133.0148	4.13	C_4_H_6_O_5_	Malic acid	[44]
**3.17**	128.0355	1.42	C_5_H_7_NO_3_	Pyroglutamic acid	[44]
**47.52**	533.3141	3.95	C_30_H_46_O_8_	Unknown	
**54.39**	531.2972	1.63	C_30_H_44_O_8_	Ganoderic acid G	[45]
**55.92**	531.2969	1.05	C_30_H_44_O_8_	Ganoderic acid G	[45]
**57.240**	529.2821	2.66	C_30_H_42_O_8_	12-Hydroxy-Ganoderic acid D	[45]
**57.906**	515.3029	2.85	C_30_H_44_O_7_	Ganoderic acid A/B	[45]
**59.223**	517.2815	1.56	C_29_H_42_O_8_	Lucidenic acid P	[46]
**60.96**	513.2856	−0.34	C_30_H_42_O_7_	Unknown	
**61.69**	513.2867	1.79	C_30_H_42_O_7_	Unknown	
**62.49**	515.3030	3.05	C_30_H_44_O_7_	Ganoderic acid A/B	[45]
**63.84**	513.2862	0.82	C_30_H_42_O_7_	Unknown	
**65.140**	515.3006	−1.6	C_30_H_44_O_7_	Ganoderic acid A/B	[45]
**65.87**	571.2921	1.47	C_32_H_44_O_9_	Ganoderic acid K	[45]
**66.72**	457.2654	−0.08	C_20_H_42_O_11_	Unknown	
**67.657**	511.2715	2.68	C_30_H_40_O_7_	Ganoderic acid D	[45]
**68.56**	513.2868	1.99	C_30_H_42_O_7_	Ganoderic acid AM1	[45]
**69.97**	511.2720	3.66	C_30_H_40_O_7_	Ganoderic acid D	[45]
**71.967**	569.2768	2.09	C_32_H_42_O_9_	12-Acetoxy-ganoderic acid F	[45]
**83.647**	265.1483	1.49	C_12_H_26_O_4_S	Dodecyl sulfate	[41]
**96.725**	309.2810	3.53	C_20_H_38_O_2_	Ethyl oleate	[41]

**Table 3:** Results of compound identification in Maitake extract using HPLC-ESI/TOF.

Rt (min.)	*m*/*z*-H	Error (ppm)	Molecular Formula	Compound	Identification
1.84	133.0144	1.14	C_4_H_6_O_5_	Malic acid	[41]
2.11	115.0039	1.88	C_4_H_4_O_4_	Fumaric acid	[41]
2.34	191.0194	−1.7	C_6_H_8_O_7_	Citric acid	[41]
3.518	382.128	2.79	C_18_H_23_O_9_	Unknown	
61.870	329.2349	4.70	C_18_H_34_O_5_	(15z)-9,12,13-Trihydroxy-15-octadecenoic acid	[41]
62.17	329.2340	1.98	C_18_H_34_O_5_	Octadecenoic acid derivatives	[41]
80.67	313.2400	4.99	C_18_H_34_O_4_	Unknown	
83.647	265.1466	−4.9	C_12_H_26_O_4_S	Dodecyl sulfate	[41]
96.725	309.2792	−2.27	C_20_H_38_O_2_	Ethyl oleate	[41]

**Table 4:** Total phenolic and flavonoid content of Maitake, Lion’s Mane and Reishi water extracts. Values are the mean of three replicate determinations ± SD.

Maitake	Lion’s Mane	Reishi
**Total phenolic compounds content [µg GAE/g DW]**		
183.75 ± 0.21	59.70 ± 0.14	13.23 ± 0.07
**Total flavonoids content [µg QE/g DW]**		
38.38 ± 0.07	13.68 ± 0.21	3.43 ± 0.03

**Maitake**

**Lion’s Mane**

**Reishi**

**Table 5:** Reducing activity of Maitake, Lion’s Mane and Reishi extracts using FRAP assay. Data are the mean ± SD of three independent experiments conducted in triplicate for each sample.

Mushroom Extract Type	μmol Trolox/g Dry Weight
**Maitake**	21.1 ± 3.2
**Lion’s Mane**	7.6 ± 1.1
**Reishi**	9.8 ± 2.4

**Table 6:** Formulation of the analyzed model washing gels.

INCI Name	Concentration [wt%]
Aqua	84.85
Sodium coco sulfate	7.00
Cocamidopropyl betaine	1.50
Coco glucoside	1.50
Glycerin	5.00
Sodium benzoate	0.50
Potassium sorbate	0.20
Sodium chloride	1.00
Lactic acid	0.45
Extract	1.00

## Data Availability

Data are contained within the article.

## Sample Availability

Not available.
